# Sustainable indoor air quality via plant-based biofiltration evaluating benzene and toluene removal efficiency and health risk reduction in pharmaceutical laboratories

**DOI:** 10.1038/s41598-026-54339-w

**Published:** 2026-06-03

**Authors:** Safinaz M. Elhadad, Shalaby ea, Ibrahim H. Saleh, Mohamed Y. Omar

**Affiliations:** 1 College of Pharmacy, Arab Academy for Science, Technology and Maritime Transport, Alexandria, Egypt; 2 Department of Environmental Studies, Institute of Graduate Studies and Research, Alexandria, Egypt; 3 Deanery of Scientific Research and Innovation, Arab Academy for Science, Technology and Maritime Transport, Alexandria, Egypt

**Keywords:** Plant-based air purification, Volatile organic compounds (VOCs), Indoor air quality (IAQ), Pharmaceutical laboratories, Green infrastructure, Health risk assessment, Chemistry, Environmental sciences

## Abstract

Chemical risks represent a significant concern in laboratory environments, especially in organic laboratories where hazardous substances such as benzene and toluene are commonly used. This study evaluates the potential health risks associated with exposure to these volatile organic compounds (VOCs) among laboratory staff and students and examines the effectiveness of plant-based biofilters (PBBFs) in improving indoor air quality (IAQ). Measurements of total VOCs, CO, CO_2_, PM_2.5_, and PM_10_ were conducted in an operational pharmaceutical laboratory using a Henan Oceanus OC-1000 multi-gas detector under both control conditions and after the introduction of indoor plants. Four ornamental species *Cordyline fruticosa*, *Syngonium podophyllum*, *Epipremnum aureum*, and *Chlorophytum comosum* were selected based on their phytoremediation potential and evaluated for their impact on IAQ. The four tested species demonstrated substantial pollutant removal, with Cordyline fruticosa achieving the highest VOC (87.5%) and CO (88.2%) reductions, *Syngonium podophyllum* achieving up to 100% reduction under controlled experimental laboratory conditions of PM_2.5_ and PM_10_, and all species showing measurable reductions in CO_2_ (20?37%). Health risk assessment confirmed that at benzene (0.3 mg/m^3^) and toluene (4 mg/m^3^) exposure levels, both cancer and non-cancer risks for staff and students remained within the U.S. EPA?s acceptable threshold (??1.0?×?10^?6^ for cancer risk; hazard quotient?<?1 for non-cancer risk), indicating that plant-based biofilters effectively mitigate laboratory air pollution while maintaining safe exposure conditions.

## Introduction

### Problem and background

Volatile organic compounds (VOCs), such as benzene and toluene, are commonly handled in organic laboratories, posing significant health risks to individuals who are regularly exposed. Toluene, which has known neurological effects, and benzene, a confirmed carcinogen, both require careful handling and strict management to minimize adverse health impacts^1^. The objective of this study is to assess the quantitative hazards of exposure to benzene and toluene and to propose mitigation strategies, including the use of indoor plants to improve indoor air quality.

### Literature review

In the late 1980s, NASA investigated the potential of common houseplants to remove VOCs from sealed environments, such as a hypothetical airtight lunar colony, marking the first demonstration of plants as effective biofilters^2^. In these studies, six common VOCs including formaldehyde, trichloroethylene, and benzene were introduced, and the plants significantly reduced their concentrations, lowering the likelihood of discomfort and adverse health effects among exposed individuals. As modern buildings become increasingly airtight, researchers have built on NASA?s findings to explore passive solutions for improving indoor air quality on Earth^3^.

It is now well recognized that VOCs are consistently present in indoor environments, especially in pharmaceutical laboratories where chemicals are frequently used over extended periods. Moreover, the composition of the surrounding matrix can influence VOC measurement and assessment. Thus, minimizing interference from the sample matrix is essential for accurate evaluation. Indoor plants offer an effective, low-maintenance solution, capable of reducing VOC concentrations by up to 50% within 24 h^4^, thereby extending the longevity and efficiency of indoor air management systems. Recent advances in volatile organic compound (VOC) mitigation technologies have highlighted the growing importance of sustainable and energy-efficient air purification strategies. Conventional VOC removal methods such as adsorption, thermal oxidation, plasma treatment, photocatalytic oxidation, and catalytic oxidation have demonstrated considerable effectiveness in reducing airborne contaminants; however, several of these approaches are associated with high operational costs, catalyst deactivation, secondary pollutant formation, and significant energy requirements^5^. Catalytic oxidation, in particular, has been recognized as one of the most efficient technologies for VOC degradation due to its high removal efficiency and ability to mineralize pollutants into less harmful compounds. Nevertheless, challenges related to catalyst stability, long-term performance, and implementation in indoor environments remain significant. In this context, plant-based biofiltration systems have emerged as an eco-friendly and sustainable complementary approach for indoor VOC remediation. Unlike conventional physicochemical technologies, phytoremediation offers additional environmental and aesthetic benefits while operating under ambient conditions with minimal energy consumption. Therefore, integrating ornamental plants into indoor environments may provide a practical and low-cost strategy for improving indoor air quality and reducing human exposure to hazardous VOCs.

Benzene is classified as a Group 1 carcinogen, indicating that it is known to cause cancer in humans, particularly by increasing the risk of leukemia with long-term exposure. In addition to its carcinogenic potential, prolonged benzene exposure can result in severe non-cancer health effects, such as bone marrow suppression and aplastic anemia. Even short-term exposure can cause adverse symptoms, including dizziness and headaches. To reduce these risks, the Environmental Protection Agency (EPA) has set a threshold limit value (TLV) for benzene at 0.1 parts per million (ppm), while the Occupational Safety and Health Administration (OSHA) has established a permissible exposure limit (PEL) of 1 ppm over an 8-h workday^6^.

Unlike benzene, toluene has not been classified as a carcinogen by the International Agency for Research on Cancer (IARC), meaning it does not carry the same level of cancer risk. However, prolonged exposure to toluene can still lead to significant health problems, including liver damage and neurological disorders. At high concentrations, its toxic effects are particularly concerning. To address these risks, the American Conference of Governmental Industrial Hygienists (ACGIH) has established a TLV for toluene at 20 ppm^7^.

Indoor air quality is a critical determinant of health and safety in enclosed environments, particularly in pharmaceutical laboratories where volatile organic compounds (VOCs) such as benzene and toluene are prevalent^8^. These compounds present serious health risks, ranging from cancer to a variety of non-cancer-related conditions^9^. Conventional air purification methods are often inadequate, which has led to growing interest in natural alternatives such as plant-based bio-filters. Indoor air pollution poses a significant threat to human health, especially in confined spaces like pharmaceutical laboratories where multiple chemical agents are routinely used. Poor IAQ has been linked to long-term health issues, including headaches, respiratory disorders, and chronic illnesses. Therefore, understanding the sources and types of indoor contaminants is essential for developing effective mitigation strategies^10^.

The health and productivity of researchers in pharmaceutical laboratories are significantly affected by exposure to volatile organic compounds (VOCs). Rationale and Objectives of this study is to evaluate the removal of VOCs due to their potential health hazards, it is equally important to recognize their broader significance in pharmaceutical laboratory settings^1^. Exposure to elevated VOC concentrations can lead to numerous symptoms, which are addressed in this section. To illustrate their relevance, the chemical structures of several common VOCs found in various environments are presented. In addition to VOCs, indoor air pollutants such as carbon monoxide (CO), carbon dioxide (CO_2_), and particulate matter (PM_2.5_ and PM_10_) were also represent critical indicators of indoor air quality and contribute to the overall assessment of the effectiveness of plant-based biofilters in laboratory environments^2^ .Understanding these structures serves as a critical reminder of the potential risks posed by indoor chemical pollutants when compared to standard workplace hygiene practices. By recognizing these hazards, laboratory personnel can be better protected through proactive and effective safety measures^11^.

### Rationale and objectives

Justification and goals of the current study explore the role of indoor plants like *Cordyline fruticosa*, *Syngonium podophyllum*, *Epipremnum aureum* (Jade Pothos), and *Chlorophytum comosum* (Spider Plant) in mitigating VOC levels, offering a natural solution to improving Indoor Air Quality and reducing health risks. Furthermore, the study will underscore the importance of adopting environmentally friendly strategies, such as phytoremediation, to mitigate the adverse effects of indoor air pollution on human health and well-being. Through meticulous analysis and reference to credible sources, this study aims to evaluate the potential of selected ornamental plants as supplementary biofiltration systems to reduce benzene and toluene exposure in pharmaceutical laboratories, while acknowledging the need for further research to validate their effectiveness under diverse conditions.

This study distinguishes itself by evaluating plant-based biofiltration under real laboratory operating conditions, rather than controlled environments, while integrating IAQ monitoring with human health risk assessment. This combined approach provides a more comprehensive and practical framework for assessing the effectiveness of phytoremediation strategies in reducing VOC exposure in occupational indoor environments.

## Materials and methods

### In-situ measurements in an operational pharmaceutical laboratory

#### IAQ parameters (VOCs, CO CO_2_, PM_2.5_ and PM_10_) without and with examined indoor plants

**Fig. 1:**
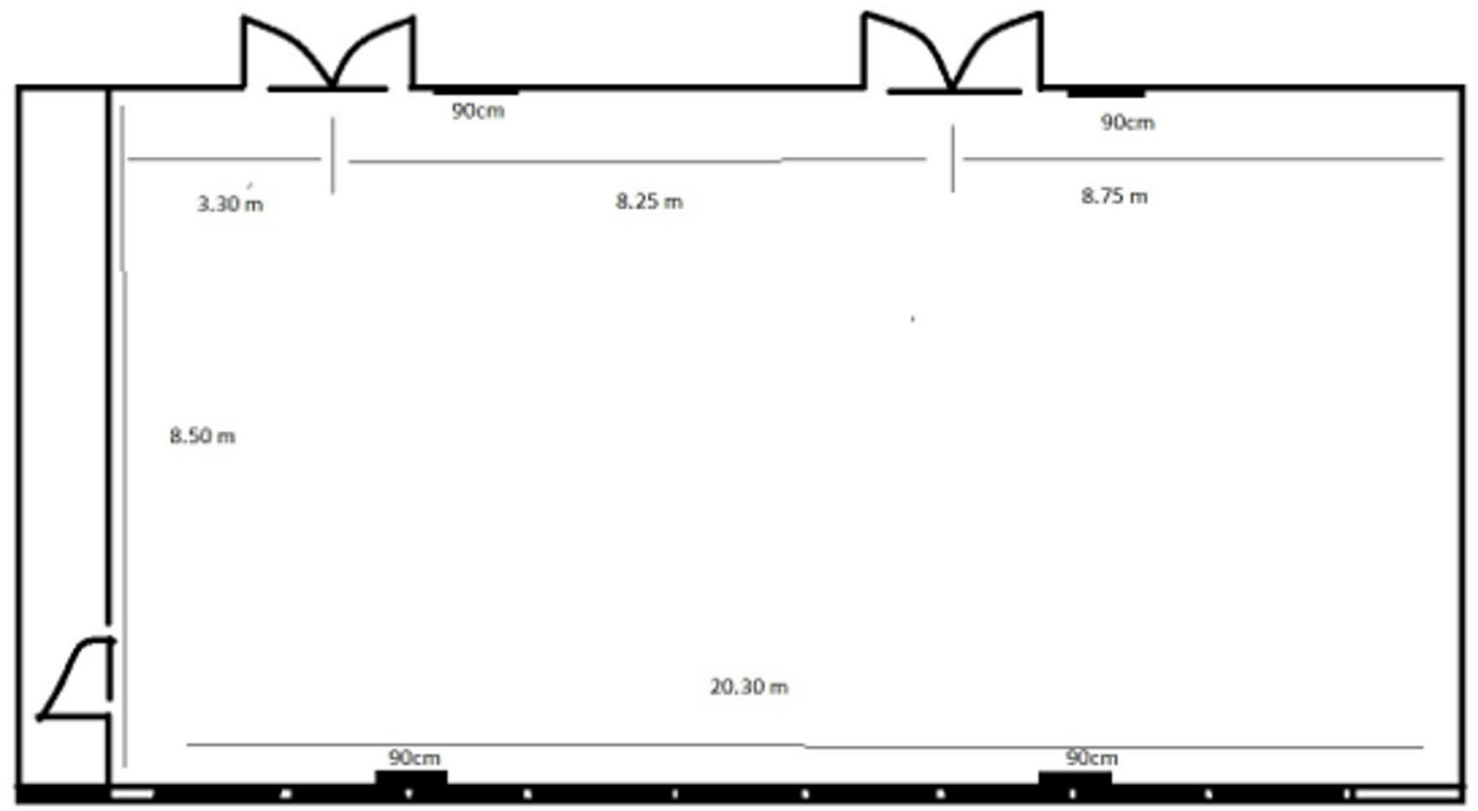
Diagram of organic chemistry laboratory.

Measurements were taken inside the organic chemistry laboratory (8.5 m width, 20.34 m length, 3.04 m height with volume: 525.59 m^3^) without and with the four examined plants. Measurements were replicated individually during students? practical section for identification of aromatic, aldehyde, and ketones identification. With all laboratory ventilation and exhaust air suction conditions and student?s number, twelve plant pots distributed on six lab benches (2 per bench/section). The measurements included the five IAQ parameters as carbon monoxide CO, carbon dioxide CO_2_, volatile organic compounds VOCs, particulate matter 2.5 (PM_2.5_) and particulate matter 10 (PM_10_) at the same time duration (40 min). The 40-min duration represents a standardized exposure time sufficient to detect measurable changes in VOC concentrations under real operating conditions using Henan OC-1000?Multi gas Detector. Taheri and Hamzehlouyan^12^ (Fig. 1).

All experiments were conducted under strictly controlled and consistent laboratory conditions to minimize environmental variability and ensure reliable comparisons. The ventilation system operated at a fixed air exchange setting with constant suction speed throughout all sessions, maintaining stable airflow conditions. Additionally, key factors influencing indoor air quality including the number of students present, the type and frequency of laboratory activities, and the use of chemicals such as benzene and toluene were kept consistent across all measurements. Although ventilation, occupancy, and other environmental factors were not separately quantified in this study, they were kept constant across all experimental conditions; therefore, the observed IAQ improvements should be interpreted as relative effects under controlled settings rather than being exclusively attributed to plant activity.

The distance between the plant-based biofiltration system and the OC-1000 monitoring device was maintained constantly to ensure measurement uniformity. Moreover, indoor lighting conditions were stable and sufficient to sustain normal plant physiological activity without fluctuations. A schematic diagram has been included in the revised manuscript to illustrate the experimental setup, including the relative positioning of the plants, monitoring instrument, and sampling area, thereby improving clarity and reproducibility. This timeframe minimizes external variability (e.g., fluctuations in occupancy, ventilation, and laboratory activities) while still allowing observation of initial phytoremediation efficiency.

The instrument was zero-calibrated according to the manufacturer?s guidelines, and span calibration was performed using certified calibration gases. The VOC detection range was 0?10 ppm with a resolution of 0.01 ppm and a detection limit of approximately 0.1 ppm. Reported accuracy for VOCs was ±?3% of the reading. CO and CO_2_ were measured in the ranges of 0?500 ppm and 0?5000 ppm, respectively, while particulate matter detection covered 0?1000 µg/m^3^.

TVOCs were measured using a PID-based OC-1000 device, which provides aggregate VOC concentrations rather than compound-specific quantification. In this study, benzene and toluene concentrations used for risk assessment were estimated based on their expected presence and relative contribution during the organic laboratory sessions involving their identification and use. Baseline measurements (in the absence of plants) were obtained prior to introducing the plant-based system under comparable environmental conditions, and each experiment was conducted in triplicate to enhance reliability and minimize random variability. All measurements were carried out according to a controlled schedule to ensure consistency in laboratory conditions across sessions. The OC-1000 device was employed as it is appropriate for real-time indoor air monitoring and occupational safety assessment.

#### Plant materials (plant morphology and physiology)

Four different indoor plants were used. Two of them are control air purifier indoor plant, which is *Epipremnum aureum* (Jade Pothos) Family: Araceae and *Chlorophytum comosum* (spider plant) Family: Asparagaceae and two tested plants, one from Araceae family which is *Syngonium podophyllum* and the other from Asparagaceae family which is *Cordyline fruticosa*.

Various parameters such as plant height, leaf area, dry weight, fresh wet weight were measured and analyzed. All plant leaves were categorized as large, medium, or small and counted. Three sample leaves were then taken from each kind and measured by leaf area software ImageJ (Java-based processing program developed at National Institutes of health and laboratory for optical and computational instrumentation). The average leaf area was then multiplied by the number of leaves counted in each species and the total surface area was calculated by adding all the individual leaf areas. Twelve potted plants from each family were used for laboratory experiments. Plants were brought from plants suppliers (college plant nursery). Loamy soil is used for pot media comprising of about 30-percent sand, 30-percent silt, 15-percent clay and 25% humus. This soil is wet, loose, with low acidity level and full nutrients suitable for growing potted plants. The plants were kept under laboratory condition for a month for acclimatization and watered whenever needed^13^.

### Probabilistic health risk assessment

The Environmental Protection Agency (EPA) respiratory health risk assessment Method has been used to assess the potential health effects of indoor volatile organic compounds on staff and student as study populations, in this study two VOCs including benzene and toluene were identified and both cancer and non-cancer risk were calculated^9^.

#### Cancer risk and non-cancer risk assessment calculations for benzene and toluene exposure inside the organic laboratory sections for staff and student

According to integrated risk information system (IRIS) database released by U.S. EPA, the potency factor (PF) which is a toxicity value that estimates the increased cancer risk from a lifetime of exposure to a chemical. It represents the slope of the dose?response curve for carcinogens and is expressed as (mg/kg-day) and reference concentration (RfC) which is an estimate of the continuous inhalation exposure to a substance that is likely to be without appreciable risk of adverse non-cancer health effects over a lifetime. It is expressed in (mg/m^3^). Variations in VOCs concentrations, exposure duration, individual inhalation rates, and laboratory operational conditions may influence calculated cancer and non-cancer risks. Table 1 was illustrated both cancer and non-cancer risk assessment, were calculated for values (0.3 mg/m^3^acceptable limit) and the max value (4 mg/m^3^) which presented during the organic practical section for both benzene and toluene^14^.

**Table 1:** Potency factor and reference concentration of VOCs.

VOCs	PF (mg kg-day)^?1^	RfC(mg m)^?3^
benzene	0.027	-
Toluene	?	5

Source: Slope factor inhalation (kg-day) mg^?1^ ? Inhalation factor for contaminant (see Table 1 in U.S. EPA (2003, 2005b, 1989)(^15^).

To calculate cancer risk for benzene using the Eqs. (1), (2):

1 $${\rm{CR = CDI }} \times {\rm{PF}}$$

2 $${\rm{CDI}}~ = \frac{{C \times CR}}{{BW}}~ \times ~\frac{{EF \times ED}}{{AT}}$$

where CR refers to Cancer Risk of benzene, CDI (chronic daily intake mg (kg/day), C is the Chemical Concentration (Obtain from sampling) mg/m^3^, CR is the Contact Rate, InhR is the inhalation rate (m^3^/day) that represents the volume of air inhaled over a specified timeframe. Long-term inhalation rates are typically expressed in units of m^3^/day. Short-term inhalation rates are typically indexed to activity levels and are expressed in units of m^3^/h or m^3^/min or (20 m^3^/day), EF is the Exposure frequency (d/y), ED is the Exposure duration (year), BW is the Body weight (kg), AT is the Averaging time (day)^16^.

To calculate non-cancer risk for toluene using Eqs. (2), (3):

3 $${\rm{NCR = CDI/RfC }}$$

where NCR refers to non-cancer risk for toluene, CDI chronic daily intake mg/(kg·day) and RfC is the reference concentration (mg m^?3^) of the inhaled non-cancerous pollutant (toluene).

### Statistical analysis

Data analysis was conducted using IBM SPSS version 20.0 (Armonk, NY: IBM Corp). Quantitative data were summarized as mean?±?standard deviation, median, and interquartile range (IQR). The significance level was set at *p*?<?0.05. For normally distributed variables, one-way ANOVA followed by Tukey?s post hoc test was used to compare differences between groups. For non-normally distributed variables, the Kruskal?Wallis test was applied as a non-parametric alternative to ANOVA because it does not assume normality of the data. Five groups were compared in total: the control (no plants) and four plant-based biofilter systems (*Cordyline fruticosa*, *Syngonium podophyllum*, *Epipremnum aureum*, and *Chlorophytum comosum*). The null hypothesis was that the median values of the outcome variables (VOC, CO, CO_2_, PM_2.5_, and PM_10_ concentrations) were equal across all groups, while the alternative hypothesis was that at least one group differed significantly. When significant differences were detected, Dunn?s multiple comparisons test was performed as a post hoc procedure to identify which groups differed. This approach ensured robust statistical comparison while accounting for potential deviations from normal distribution.

## Results and discussion

### In-situ measurements in an operational pharmaceutical laboratory

#### Measurement of organic lab VOCs

**Fig. 2:**
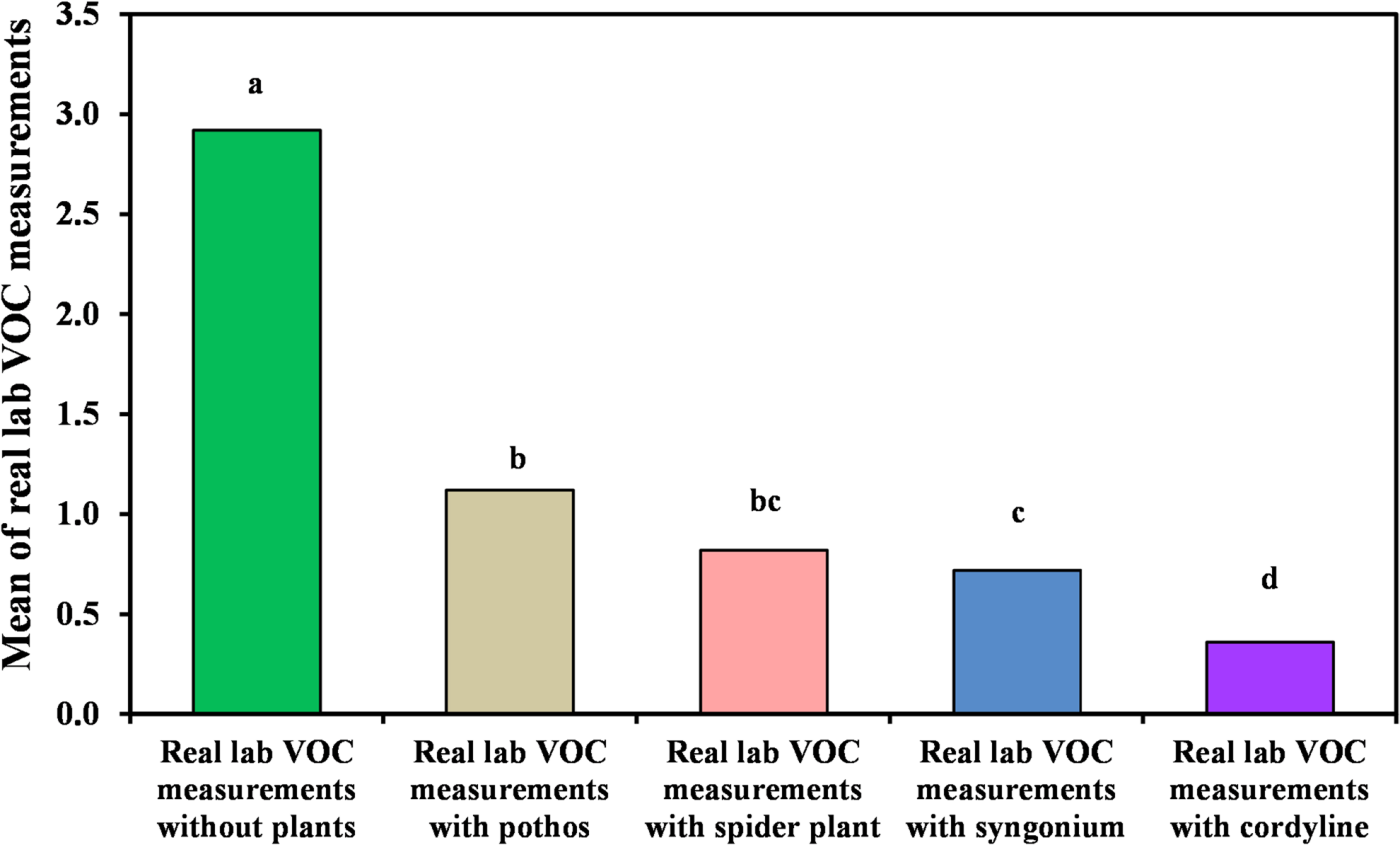
Comparison between real lab VOCs measurements with and without examined plant.

**Fig. 3:**
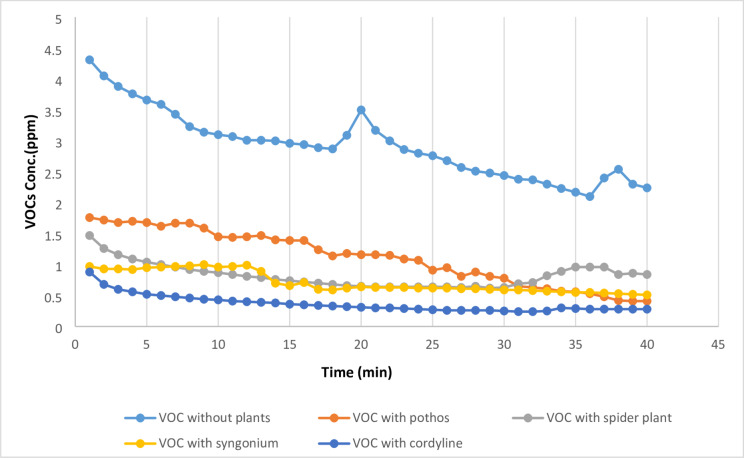
Real laboratory measurements of organic lab VOCs without and with the four examined plants.

The efficiency of the investigated plant species in reducing volatile organic compounds (VOCs) in the organic laboratory environment is summarized in Table 2 and illustrated in Figs. 2 and 3. Among the evaluated species, *Cordyline fruticosa* demonstrated the greatest ability to reduce VOC concentrations, achieving a removal efficiency of 87.5%. The statistical analysis shows significant differences (*p*?<?0.001) among the VOC measurements with different plant species. The letters ?a,? ?b,? ?c,? and ?d? indicate statistically significant differences between groups, with ?a? being the highest and ?d? the lowest VOC levels. The average concentration decreased from 2.92?±?0.54 ppm prior to treatment to 0.36?±?0.14 ppm following exposure to the plant-based biofilter. *Syngonium podophyllum* ranked second, exhibiting a VOC removal efficiency of 81.69%, with concentrations reduced to 0.72?±?0.17 ppm. Moderate reductions were observed for *Epipremnum aureum*, which achieved a removal efficiency of 77.23%. In contrast, *Chlorophytum comosum* showed comparatively lower performance, with a removal efficiency of 62.5%. These findings highlight notable interspecies variation in VOC mitigation capacity within the laboratory setting.

**Table 2:** Comparison between real lab VOCs measurements with and without examined plant.

Real lab VOC measurements	Real lab VOC measurements without plants (*n*?=?40)	Real lab VOC measurements with pothos (*n*?=?40)	Real lab VOC measurements with *Chlorophytum comosum* (*n*?=?40)	Real lab VOC measurements with syngonium (*n*?=?40)	Real lab VOC measurements with *Cordyline fruticosa* (*n*?=?40)	H	*p*
Min.?Max.	2.10?4.31	0.41?1.76	0.62?1.47	0.51?1.0	0.24?0.88		
Mean?±?SD.	2.92^a^?±?0.54	1.12^b^?±?0.44	0.82^bc^?±?0.19	0.72?+?±?0.17	0.36^d^?±?0.14	152.82*	<?0.001*
Median	2.92	1.16	0.80	0.63	0.31		
IQR	2.5?3.2	0.72?1.5	0.65?0.94	0.59?0.93	0.28?0.42		

IQR, Inter quartile range; SD, Standard deviation.

H: H for Kruska?Wallis test, pairwise comparison bet. Each 2 groups were done using Post Hoc Test (Dunn?s for multiple comparisons test).

p: p value for comparing between the three different studied time.

*Statistically significant at *p*???0.05.

#### Measurement of organic lab carbon monoxide

**Fig. 4:**
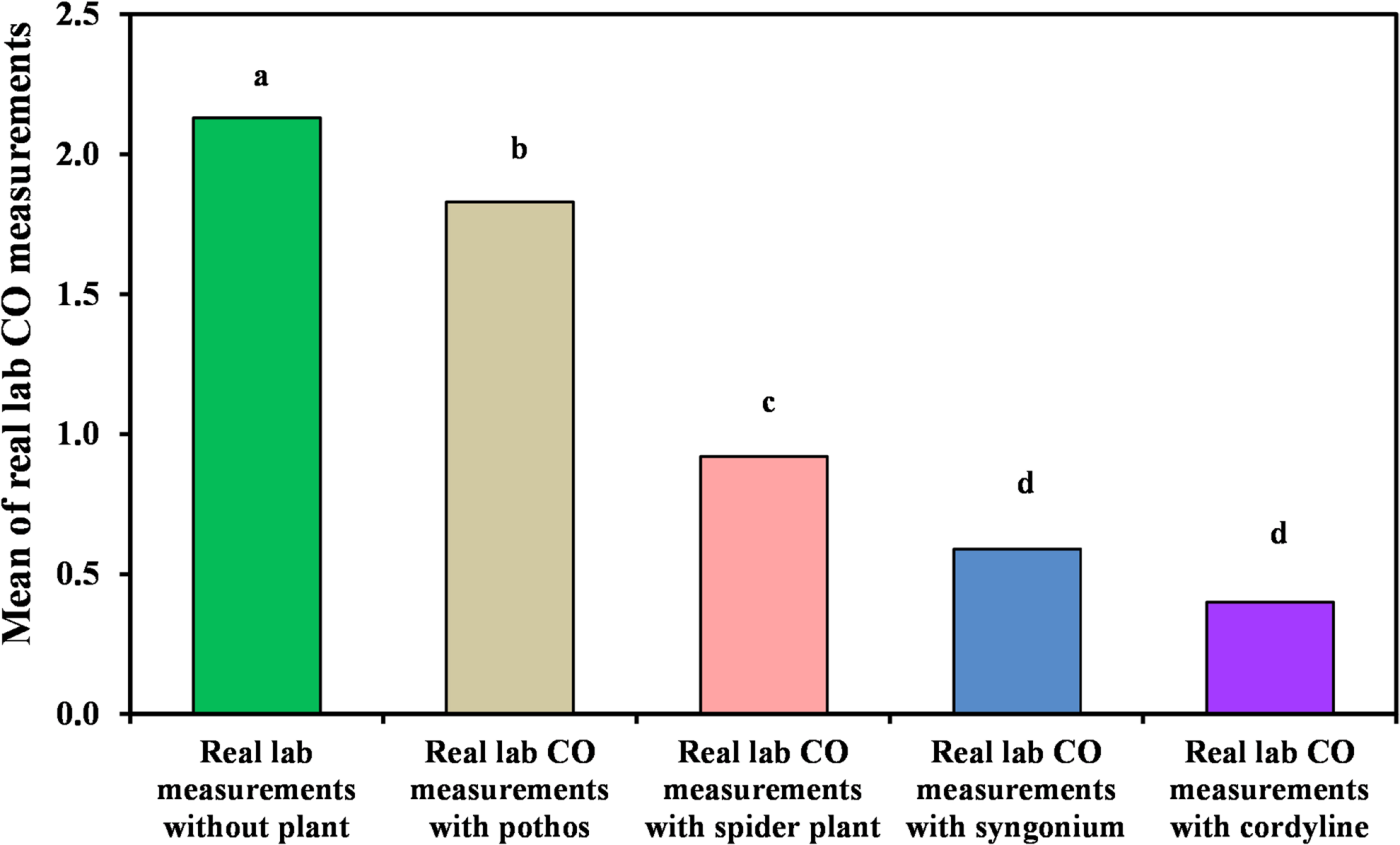
Comparison between real lab CO measurements with and without examined plant.

**Fig. 5:**
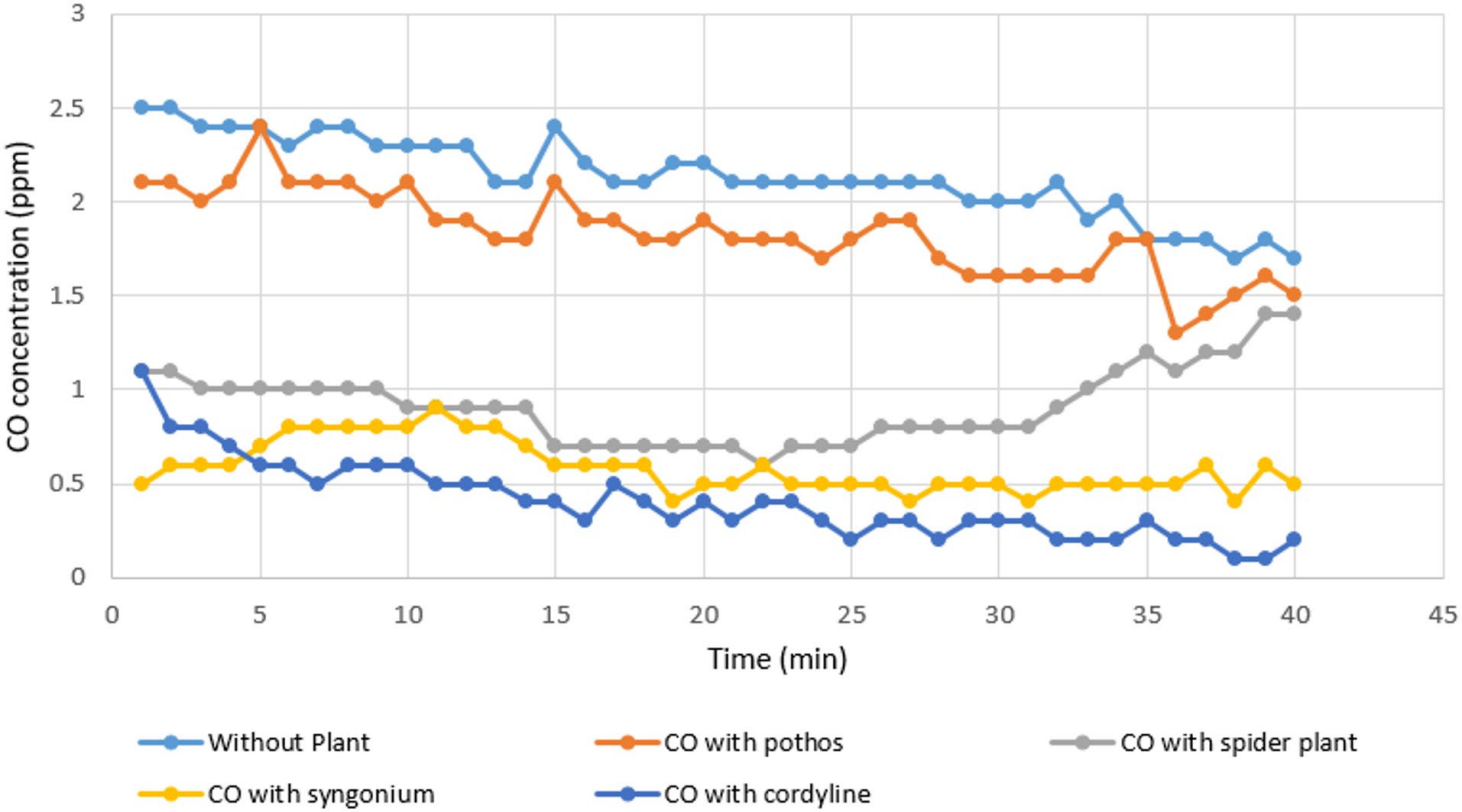
Real laboratory measurements of organic lab CO without and with the four examined plants.

Changes in indoor carbon monoxide levels following the introduction of plant-based biofilters are presented in Table 3; Figs. 4 and 5. The results indicate that *Cordyline fruticosa* was the most effective species in reducing CO concentrations over time, achieving a removal efficiency of 88.23% and a final mean concentration of 0.40?±?0.21 ppm. The second highest reduction was recorded for *Syngonium podophyllum*, which achieved a removal efficiency of 70.58% with a mean concentration of 0.59?±?0.13 ppm. Lower reductions were observed for *Chlorophytum comosum* and *Epipremnum aureum*, which both showed removal efficiencies of approximately 11.7%, resulting in mean concentrations of 0.92?±?0.20 ppm and 1.83?±?0.23 ppm, respectively.

**Table 3:** Comparison between real lab CO measurements with and without examined plant.

Real lab CO measurements	Real lab measurements without plant (*n*?=?40)	Real lab CO measurements with pothos (*n*?=?40)	Real lab CO measurements with Chlorophytum comosum (*n*?=?40)	Real lab CO measurements with Syngonium (*n*?=?40)	Real lab CO measurements with *Cordyline fruticosa* (*n*?=?40)	H	*p*
Min.Max.	1.70?2.50	1.30?2.40	0.60?1.40	0.40?0.90	0.10?1.10		
Mean?±?SD.	2.13^a^?±?0.22	1.83^b^?±?0.23	0.92^c^?±?0.20	0.59^d^?±?0.13	0.40^d^?±?0.21	173.43*	<?0.001*
Median	2.10	1.80	0.90	0.55	0.35		
IQR	2.0?2.3	1.7?2.0	0.70?1.0	0.50?0.65	0.25?0.50		

IQR, Inter quartile range; SD, Standard deviation.

H: H for Kruskal?Wallis test, Pairwise comparison bet. Each 2 groups were done using Post Hoc Test (Dunn?s for multiple comparisons test).

p: p value for comparing between the three different studied time.

*Statistically significant at *p*???0.05.

#### Measurement of organic lab carbon dioxide

**Fig. 6:**
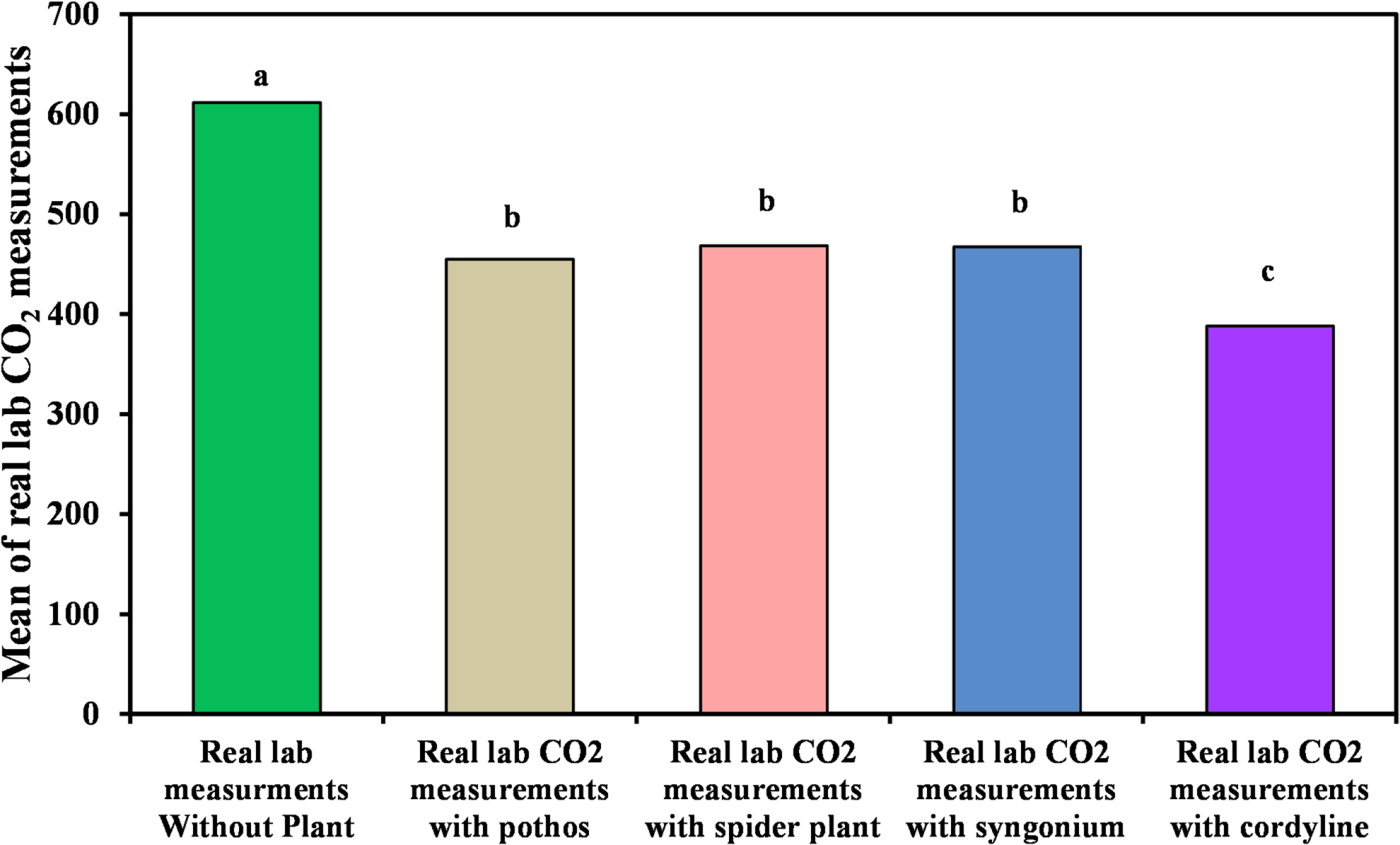
Comparison between real lab CO_2_ measurements with and without examined plant.

**Fig. 7:**
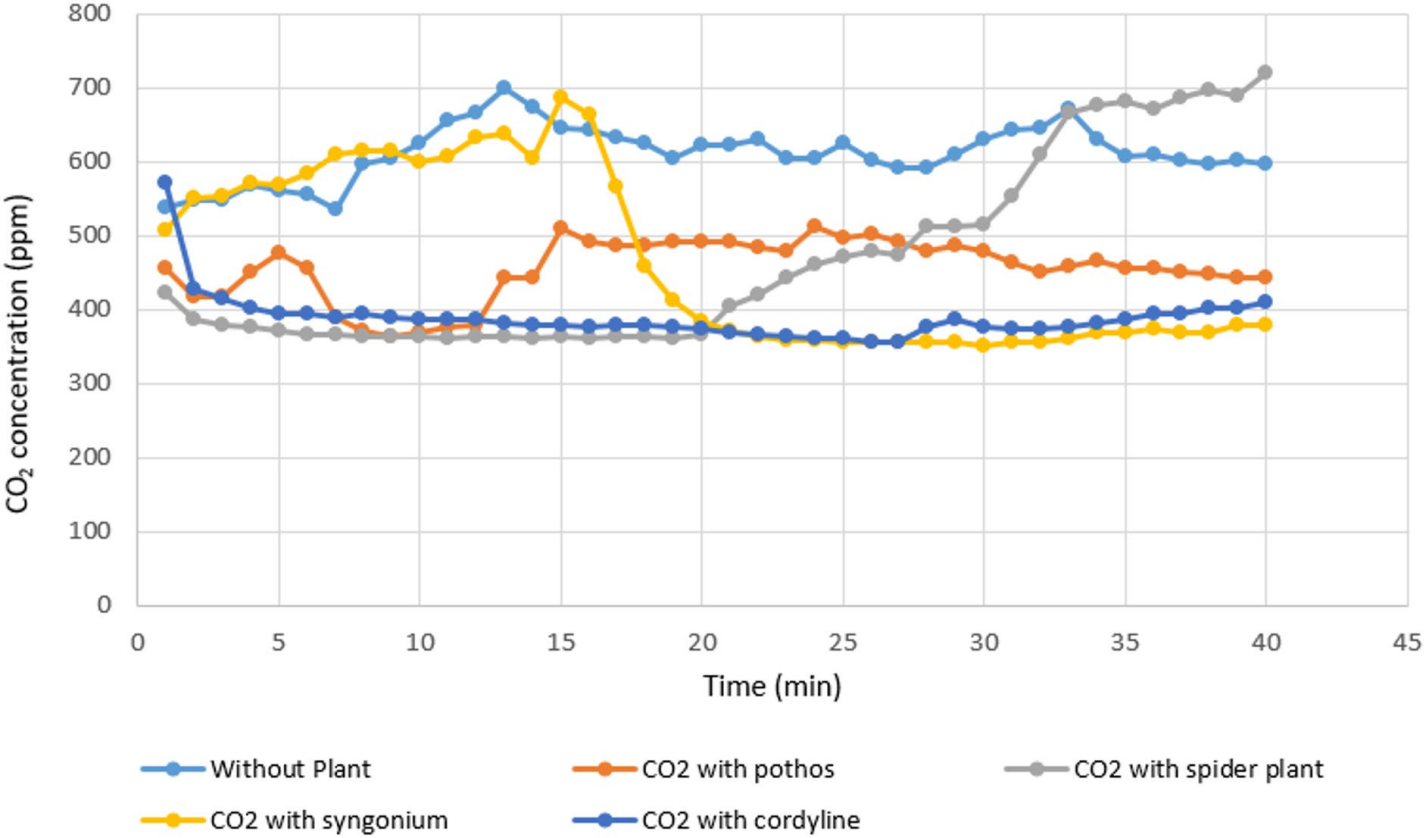
Real laboratory measurements of organic lab CO_2_ without and with the four examined plants.

The influence of the tested plants on indoor carbon dioxide concentrations is presented in Table 4; Figs. 6 and 7. Among the studied species, *Cordyline fruticosa* again exhibited the highest reduction capability, with a CO_2_ removal efficiency of 36.78%, resulting in an average concentration of 388.2?±?33.73 ppm. The next highest reductions were observed for *Syngonium podophyllum*, *Chlorophytum comosum*, and *Epipremnum aureum*, which achieved removal efficiencies of 31.27%, 25.91%, and 20.2%, respectively. The corresponding mean concentrations were 467.5?±?119.0 ppm, 468.4?±?126.2 ppm, and 455.2?±?41.19 ppm. Overall, while all plant species contributed to measurable decreases in CO_2_ levels, the reductions were less pronounced compared with those observed for VOCs and CO.

**Table 4:** Comparison between real lab CO_2_ measurements with and without examined plant.

Real lab CO_2_ measurements	Real lab measurements without plant (*n*?=?40)	Real lab CO_2_ measurements with pothos (*n*?=?40)	Real lab CO_2_ measurements with *Chlorophytum comosum* (*n*?=?40)	Real lab CO_2_ measurements with Syngonium (*n*?=?40)	Real lab CO_2_ measurements with *Cordyline fruticosa* (*n*?=?40)	H	*p*
Min.?Max.	535.0?700.0	363.0?513.0	362.0?719.0	352.0?688.0	356.0?572.0		
Mean?±?SD.	611.8^a^?±?37.87	455.2^b^?±?41.19	468.4^b^?±?126.2	467.5^b^?±?119.0	388.2^c^?±?33.73	72.415*	<?0.001*
Median	37.87	457.0	412.5	381.0	382.0		
IQR	597.5?632.0	443.0?488.0	363.0?534.5	360.0?592.0	375.0?394.0		

IQR, Inter quartile range; SD, Standard deviation.

H: H for Kruskal?Wallis test, pairwise comparison bet. Each 2 groups were done using Post Hoc Test (Dunn?s for multiple comparisons test).

p: p value for comparing between the three different studied time.

#### Measurement of organic lab PM_2.5_

**Fig. 8:**
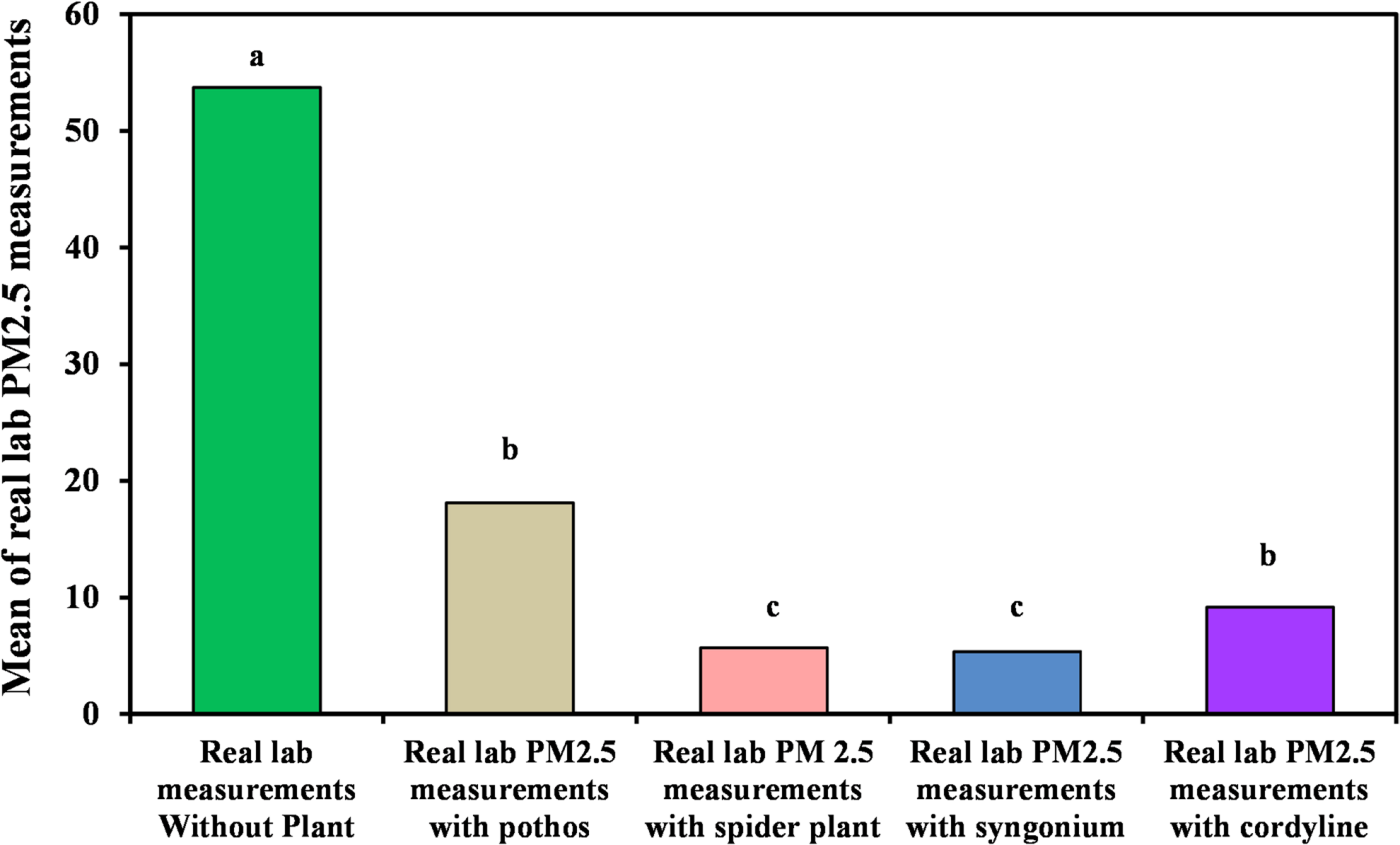
Comparison between real lab PM_2.5_ measurements with and without examined plant.

**Fig. 9:**
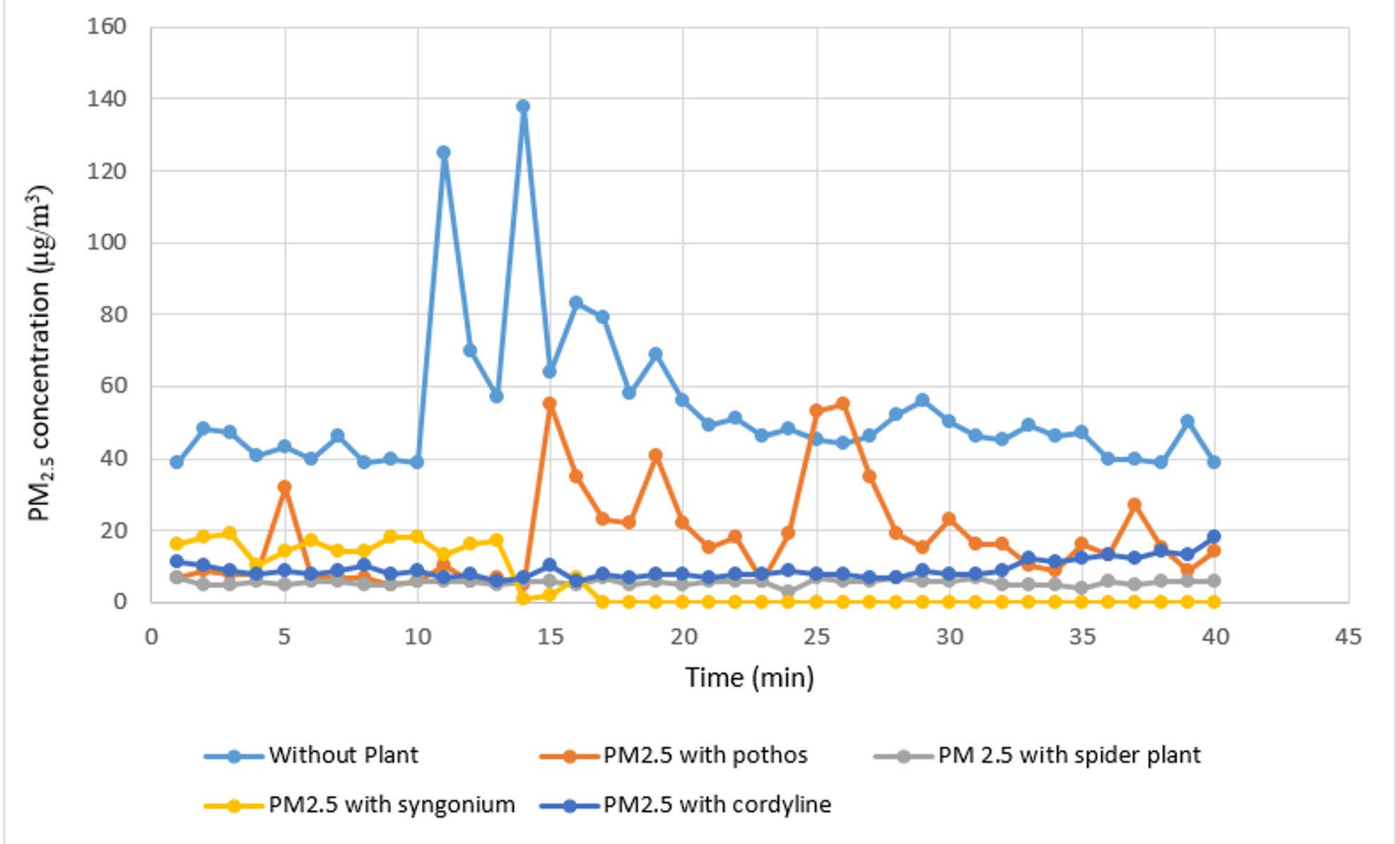
Real laboratory measurements of organic lab PM_2.5_ without and with the four examined plants.

The removal efficiency of the examined plant species for PM_2.5_ particles is summarized in Table 5 and depicted in Figs. 8 and 9. *Syngonium podophyllum* exhibited the most significant effect, achieving up to 100% reduction under controlled experimental laboratory conditions of PM_2.5_ during the monitoring period, with a mean concentration of 5.35?±?7.49 µg/m^3^. *Chlorophytum comosum* ranked second, demonstrating a removal efficiency of 84.61%, followed by *Epipremnum aureum*, which achieved 64.10% removal. The lowest reduction was recorded for *Cordyline fruticosa*, with a removal efficiency of 53.84% and a mean concentration of 18.12?±?13.83 µg/m^3^. These results indicate that *Syngonium podophyllum* is particularly effective for fine particulate matter removal.

**Table 5:** Comparison between real lab PM_2.5_ measurements with and without examined plant.

Real lab PM2.5 measurements	Real lab measurements without plant (*n*?=?40)	Real lab PM_2.5_ measurements with pothos (*n*?=?40)	Real lab PM_2.5_ measurements with *Chlorophytum comosum* (*n*?=?40)	Real lab PM_2.5_ measurements with Syngonium (*n*?=?40)	Real lab PM_2.5_ measurements with *Cordyline fruticosa* (*n*?=?40)	H	*p*
Min.?Max.	39.0?138.0	5.0?55.0	3.0?7.0	0.0?19.0	6.0?18.0		
Mean?±?SD.	53.73^a^?±?21.01	18.12^b^?±?13.83	5.68^c^?±?0.83	5.35^c^?±?7.49	9.18^b^?±?2.43	132.10*	<?0.001*
Median	47.0	15.0	6.0	0.0	8.0		
IQR	42.0?56.0	7.5?22.5	5.0?6.0	0.0?14.0	8.0?10.0		

IQR, Inter quartile range; SD, Standard deviation.

H: H for Kruskal?Wallis test, pairwise comparison bet. Each 2 groups were done using Post Hoc Test (Dunn?s for multiple comparisons test).

p: p value for comparing between the three different studied time.

*Statistically significant at *p*???0.05.

#### Measurement of organic lab PM_10_

**Fig. 10:**
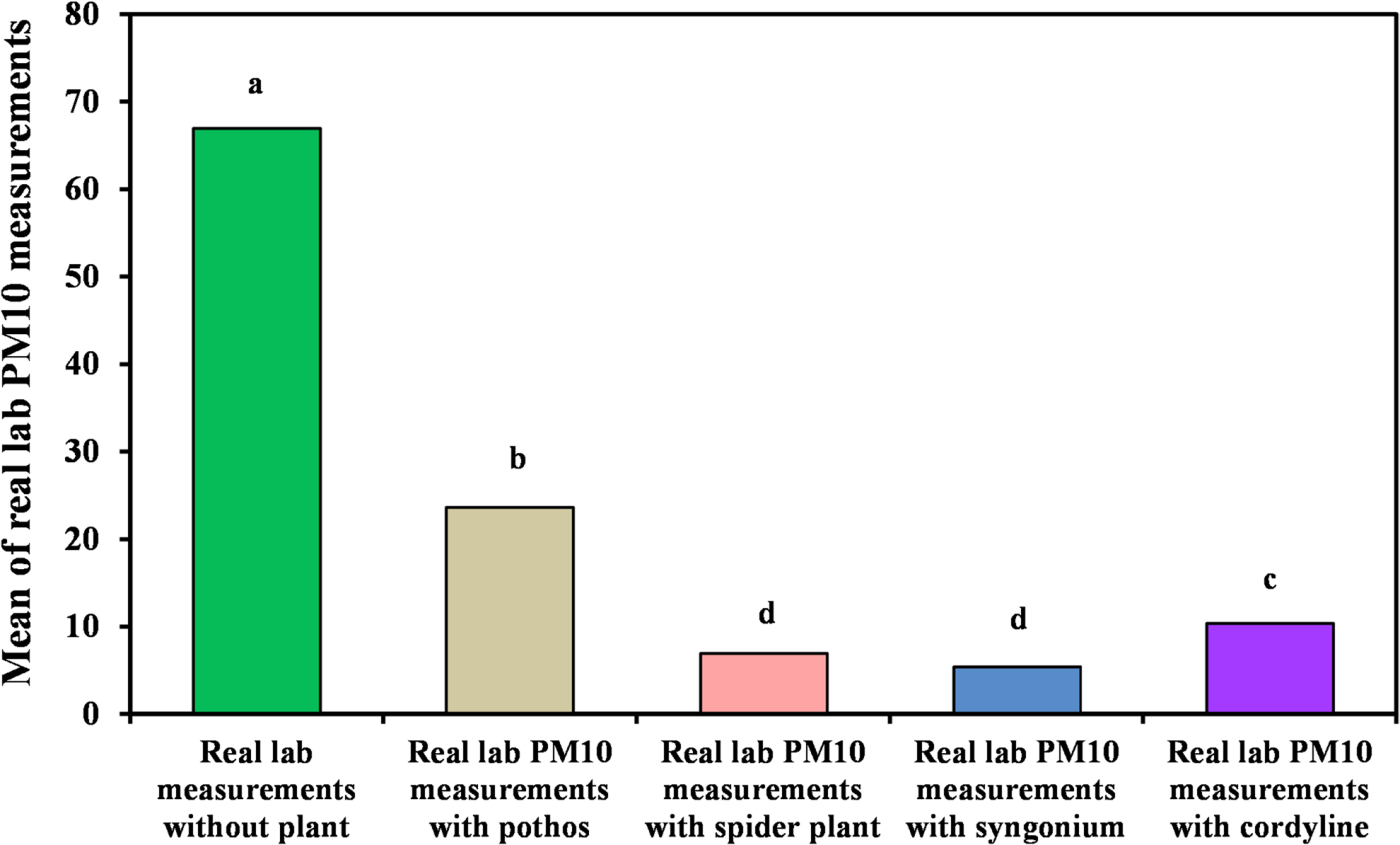
Comparison between real lab PM_10_ measurements with and without examined plant.

**Fig. 11:**
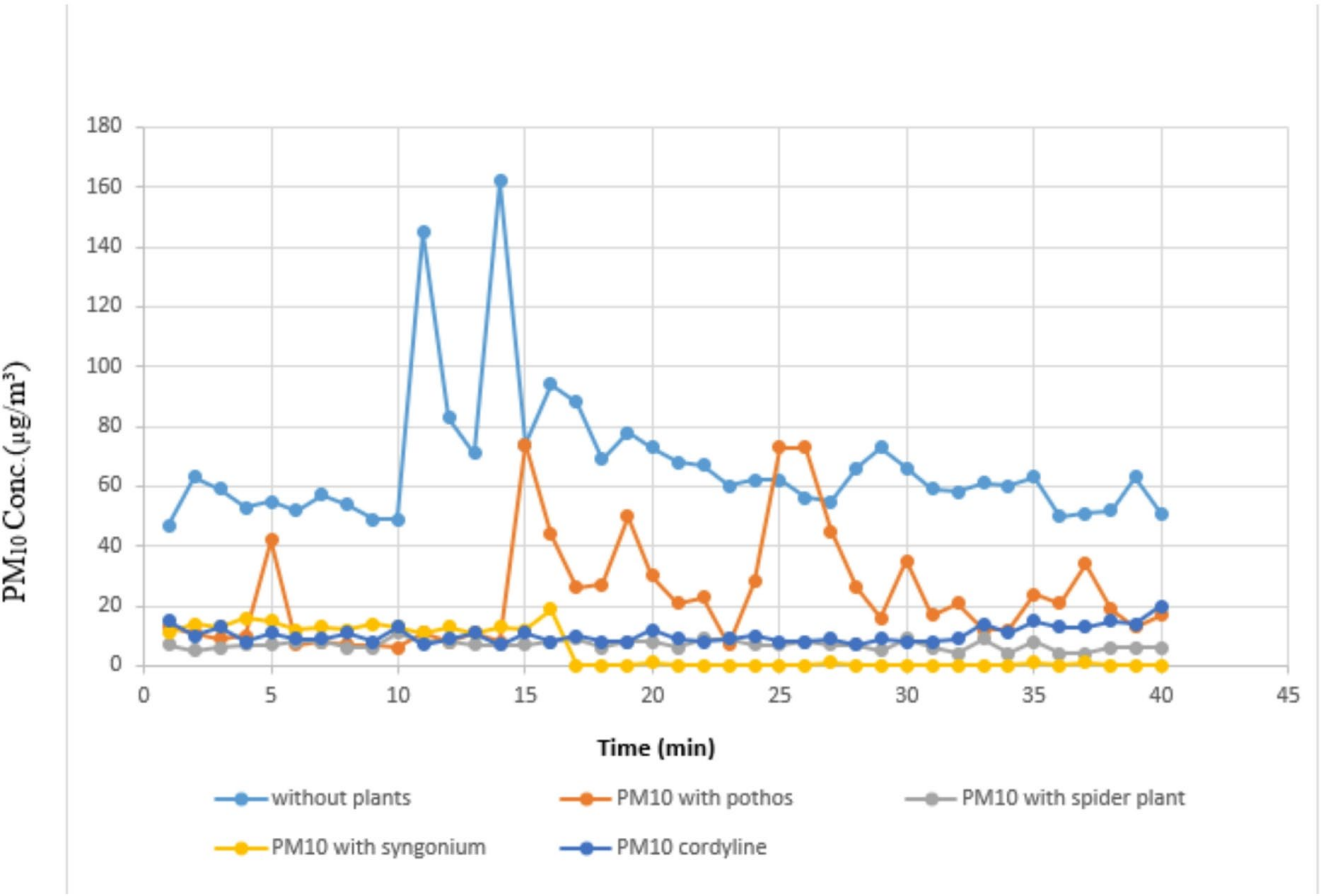
Real laboratory measurements of organic lab PM_10_ without and with the four examined plants.

Table 6; Figs. 10 and 11 present the changes in PM_10_ concentrations in the laboratory environment following plant introduction. Consistent with the PM_2.5_ results, *Syngonium podophyllum* achieved the highest reduction efficiency, reaching 100% removal with a mean concentration of 5.40?±?6.62 µg/m^3^. *Chlorophytum comosum* exhibited the second-highest performance with a removal efficiency of 88.23%, followed by *Epipremnum aureum* (66.66%) and *Cordyline fruticosa* (60.78%). These results further emphasize the superior particulate capture capability of *Syngonium podophyllum* among the tested plant species.

**Table 6:** Comparison between real lab PM_10_ measurements with and without examined plant.

Real lab PM10 measurements	Real lab measurements without plant (*n*?=?40)	Real lab PM_10_ measurements with pothos (*n*?=?40)	Real lab PM_10_ measurements with *Chlorophytum comosum* (*n*?=?40)	Real lab PM_10_ measurements with Syngonium (*n*?=?40)	Real lab PM_10_ measurements with *Cordyline fruticosa* (*n*?=?40)	H	*p*
Min.?Max.	47.0?162.0	6.0?74.0	4.0?11.0	0.0?19.0	7.0?20.0		
Mean?±?SD.	66.95^a^?±?22.88	23.65^b^?±?18.50	6.95^d^?±?1.58	5.40^d^?±?6.62	10.38^c^?±?2.87	129.83*	<?0.001*
Median	61.50	18.0	7.0	0.50	9.0		
IQR	54.5?70.0	10.5?29.0	6.0?8.0	0.0?12.5	8.0?12.5		

IQR, Inter quartile range; SD, Standard deviation.

H: H for Kruskal?Wallis test, pairwise comparison bet. Each 2 groups were done using Post Hoc Test (Dunn?s for multiple comparisons test).

p: p value for comparing between the three different studied time *Statistically significant at *p*???0.05.

### Plant morphology and physiology measurements

Morphological and physiological attributes of the four evaluated plant species *Epipremnum aureum*, *Chlorophytum comosum*, *Syngonium podophyllum*, and *Cordyline fruticosa* are summarized in Table 7. Plant height showed substantial variation among species, ranging from 17 cm for Epipremnum aureum to 59 cm for Cordyline fruticosa. Measurements of fresh biomass (three leaves) differed between species, reflecting variations in leaf morphology and water content. In agreement with this, Torpy et al.^17^ reported that plant species with greater leaf area and biomass generally exhibit higher pollutant removal efficiency due to increased surface deposition and enhanced stomatal uptake capacity, supporting the role of plant morphological traits in improving indoor air quality. In line with recent findings, Wang et al.^18^ reported that indoor ornamental plants with higher leaf biomass and stomatal density demonstrate enhanced particulate matter capture and VOC uptake efficiency, emphasizing that structural plant traits play a critical role in phytoremediation performance. Similarly, dry biomass values indicated that *Chlorophytum comosum* and *Cordyline fruticosa* possessed the highest dry matter content among the evaluated plants. These morphological and physiological differences may contribute to the observed variation in pollutant removal performance.

**Table 7:** Morphological parameter for examined plants.

	Epipremnum aureum	*Chlorophytum comosum*	*Syngonium podophyllum*	*Cordyline fruticosa*
Plant height	17 cm	26 cm	55 cm	59 cm
Fresh wet weight of three leaves	4.6392 gm	7.3361 gm	4.7537 gm	3.6242 gm
Dry weight of three leaves	0.3455 gm	0.6610 gm	0.6838 gm	0.8025 gm

### Risk assessment calculations for benzene and toluene exposure inside the organic laboratory sections

Health risk calculations associated with exposure to benzene and toluene within the organic laboratory are presented in Tables 8, 9 and 10. The assessment considered both cancer risk (benzene) and non-cancer risk (toluene) using standard exposure parameters. The exposure scenario assumed that laboratory staff experienced 100 exposure days per year over a 5-year period, whereas students were assumed to attend 10 practical laboratory sessions per year over a 2-year period. Additional parameters included body weights of 90 kg for staff and 70 kg for students, and averaging times of 40 years (14,600 days) for staff and 20 years (7300 days) for students.

**Table 8:** Risk assessment calculations for benzene and toluene exposure inside the organic laboratory sections.

	Staff	Student
CR = contact rate?=?Inhalation rate (m^3^/day)	20	20
EF (d/y)	100 d/y	10 d/y
ED (year)	5 y	1 y
BW (kg)	90 kg	70 kg
AT (day)	40 y?=?14,600 days	20 y?=?7300 days
CDI: Concentration: 0.3 mg/m^3^ (Acceptable Exposure Limit)	0.3?×?20?×?100?×?5/90?×?14,600 = 0.002283105 mg/(kg×day)	0.3?×?20?×?10?×?1/70?×?7300 = 0.000117417 mg/(kg×day)
For conc. 0.3 mg/m^3^ PF for benzene?=?0.027 Cancer Risk?=?CDI× PF for benzene	0.002283105?×?0.027 = 6.16438?×?10^??5^ Acceptable risk	0.000117417?×?0.027 = 3.17025?×?10^??6^ Acceptable risk
For conc. 0.3 mg/m^3^ RfC for toluene?=?5 Non-Cancer Risk?=?CDI/RfD for toluene	0.002283105/5 = 0.000456621 Acceptable risk	0.000117417/5 =2.34834?×?10^??5^ Acceptable risk
CDI: Concentration: 4 mg/m^3^ (Highest Exposure Limit in the Lab.)	4?×?20?×?100?×?5/90?×?14,600 = 0.0304414 mg/(kg×day)	4?×?20?×?10?×?1/70?×?7300 =0.001565558 mg/(kg×day)
For conc. 4mg/m^3^ PF for Benzene?=?0.027 Cancer Risk?=?CDI×PF for benzene	0.0304414?×?0.027 =0.000821918 Acceptable risk	0.001565558?×?0.027 = 4.22701?×?10^??5^ Acceptable risk
For conc. 4 mg/m^3^ RfD for Toluene?=?5 Non-Cancer Risk?=?CDI/RfD for toluene	0.0304414/5 = 0.00608828 Acceptable risk	0.001565558/5 = 0.000313112 Acceptable risk

**Table 9:** Risk assessment for exposure of staff to benzene and toluene.

Significant VOC	Staff			
	Benzene		Toluene	
Concentration mg/m^3^	0.3	4	0.3	4
CDI mg/(kg×day)	2.30.002283105	0.0304414	2.30.002283105	0.0304414
PF	0.027		?	
Inhalation RfD mg/m^3^	?		5	
Cancer risk (CDI×PF)	6.16438?×?10^??5^	0.000821918	?	?
Cancer risk assessment	Risk?<?1 Acceptable risk	Risk?<?1 Acceptable risk	?	?
Non-cancer risk (CDI/RfC)	?	?	0.000456621	0.00608828
Non cancer risk assessment	?	?	Risk?<?1 Acceptable risk	Risk?<?1 Acceptable risk

**Table 10:** Risk assessment for exposure of student to benzene and toluene.

Significant VOC	Student			
	Benzene		Toluene	
Concentration mg/m^3^	0.3	4	0.3	4
CDI mg/(kg×day)	0.000117417	0.001565558	0.000117417	0.001565558
PF	0.027		?	
Inhalation RfD mg/m^3^	?		5	
Cancer risk (CDI×PF)	3.17025?×?10^??6^	4.22701?×?10^??5^	?	?
Cancer risk assessment	Risk?<?1 acceptable risk	Risk?<?1 acceptable risk	?	?
Non-cancer risk (CDI/RfC)	?	?	2.34834?×?10^??5^	0.000313112
Non cancer risk assessment	?	?	Risk?<?1 Acceptable risk	Risk?<?1 Acceptable risk

Risk calculations were performed using measured pollutant concentrations of 0.3 mg/m^3^ for benzene and 4 mg/m^3^ for toluene. The results indicated that exposure to 0.3 mg/m^3^ benzene resulted in acceptable cancer risk levels for both staff and students. Similarly, exposure to 4 mg/m^3^ toluene produced non-cancer risk values within acceptable limits.

According to risk assessment guidelines, a cancer risk value???1.0?×?10^?6^ is considered negligible, whereas risk levels approaching 1.0?×?10?^3^ indicate potential concern. A threshold of 1.0?×?10^?4^ is commonly regarded as an acceptable risk level^16^. The calculated values in the present study remained below these critical thresholds.

The relatively low risk estimates can be attributed to differences in exposure patterns between staff and students. While staff members were assumed to experience longer exposure durations, students participated in a limited number of laboratory sessions annually. Furthermore, the measured benzene and toluene concentrations remained below the recommended exposure limits established by the U.S. Environmental Protection Agency (EPA) and occupational exposure guidelines.

Overall, the findings demonstrate that the implementation of plant-based biofilters significantly improved indoor air quality in the pharmaceutical laboratory. Among the investigated species, *Cordyline fruticosa* exhibited the greatest efficiency in reducing VOCs and carbon monoxide, whereas *Syngonium podophyllum* proved most effective for the removal of PM_2.5_ and PM_10_. All four plant species contributed to reductions in carbon dioxide concentrations, although the magnitude of improvement was smaller compared with other pollutants.

The health risk assessment further confirmed that exposure to benzene and toluene remained within acceptable limits for both laboratory staff and students. These results support the application of plant-based biofiltration systems as a sustainable and practical approach for mitigating indoor air pollution, particularly when combined with appropriate ventilation strategies and the use of personal protective equipment (PPE).

### Discussion

Our findings align closely with previous studies demonstrating the ability of ornamental plants to reduce indoor VOC concentrations. *Cordyline fruticosa* removed organic laboratory volatile organic compounds (VOCs) with an efficiency of 87.5% greater than that of *Epipremnum aureum* (81.69%). *Chlorophytum comosum* 62.5%), which is in line with experimental evidence showing that *Epipremnum aureum* had a greater benzene removal rate than *Chlorophytum comosum*^19, 20^, han. While a small number of studies (Wood et al.^21^, Pettit et al.^22^ have demonstrated the effectiveness of potted plants in reducing the concentration of volatile organic compounds (VOCs) in virtually sized rooms, very few studies have examined the indoor quality of university pharmaceutical laboratories^23^. Prior research ^24^, ^25^,^26^, ^27^, ^28^, ^29^ has assessed the potential of indoor potted plants to lower VOCs. The potential of potted plants to reduce VOC concentrations in rooms or chambers was assessed, in accordance with the results of the 2019 study by Pettit, Irga, et al. Another study looked at how volatile organic compounds (VOCs) were controlled between 20 and 300 parts per billion (ppb)^30^and the decrease in VOC concentrations in ketones, acetone, methyl ethyl ketone, methyl isobutyl ketone, and BTEX (benzene, toluene) (Torpy et al.^17^). A few colleges in Eastern Canada have employed this cutting-edge technology because they find it intriguing for improving indoor air quality in buildings^31^. Previous research by Wood et al.^21^and Pettit et al. ^22^supports the effectiveness of potted plants in reducing VOC concentrations. Our results are consistent with these findings, showing substantial VOC reduction in the laboratory environment. The CO_2_, CO, PM_2.5_, and PM_10_ levels were maintained within permissible limits after introducing the plants, confirming the efficiency of PBBFs in enhancing IAQ. These outcomes are corroborated by studies from Irga et al.^26^ and Ibrahim et al. ^32^, which reported similar results using plant-based bio-filter systems or active green walls (AGWs).

The present study also observed that levels of CO_2_, CO, PM_2.5_, and PM_10_ remained within permissible limits after implementing plant-based bio-filter systems (PBBFs). This supports earlier work by Irga et al.^26^ and Ibrahim et al.^32^, who reported comparable results using PBBFs and active green walls (AGWs). According to recent research, combining a plant-based biofilter with ventilation can lower levels of TVOC, CO_2_, CO, PM_2.5_, and PM_10_ by 87.5%, 88.23%, 36.78%, up to 100%, respectively; reduction under controlled experimental laboratory conditions. The results support research by Shambhavi Sharma et al.^33^ that suggests a range of indoor potted plants provide credence to the idea that plant-based bio-filters can effectively improve indoor air quality^34^.

The findings of the current study align with recent literature emphasizing the need for sustainable and low-energy VOC mitigation technologies for indoor environments. According to Tiwari et al.^5^, although catalytic oxidation and other advanced VOC removal technologies exhibit high degradation efficiencies, their practical application may be limited by operational complexity, energy demand, catalyst poisoning, and maintenance costs. In contrast, plant-based biofiltration systems represent a biologically driven and environmentally sustainable alternative capable of operating continuously under normal indoor conditions. However, possible confounding factors such as variations in laboratory ventilation, occupancy patterns, and fluctuations in pollutant emissions must be acknowledged, as they may have influenced pollutant concentrations alongside plant uptake. Despite these uncertainties, the consistency of our findings with earlier studies provides strong evidence for the role of plants in improving indoor air quality (IAQ).

The health risk assessment carried out in this study further supports the effectiveness of current laboratory practices. According to EPA guidelines, a cancer risk of less than or equal to 1 in 1,000,000 (1.0?×?10^?6^) is considered acceptable. Our calculations confirmed that the cancer risk associated with benzene exposure remained well below this threshold, even at higher exposure doses. The non-cancer risks for toluene were also within safe limits, suggesting that neurological and other adverse health effects are unlikely under present laboratory conditions. These outcomes reflect the adequacy of existing control measures, including proper ventilation and the use of personal protective equipment (PPE).

Moreover, the calculated Chronic Daily Intake (CDI) values for benzene and toluene were within the U.S. EPA reference concentration (RfC) limits, and the measured exposure concentrations did not exceed the threshold limit values (TLVs) or time-weighted averages (TWAs) established for occupational safety. This contrasts with the findings of Marki? (2023), who reported that landfill workers exposed to benzene, toluene, and xylene experienced exposure levels above recommended standards, indicating greater health risks in those occupational settings compared with our controlled laboratory environment.

**Fig. 12:**
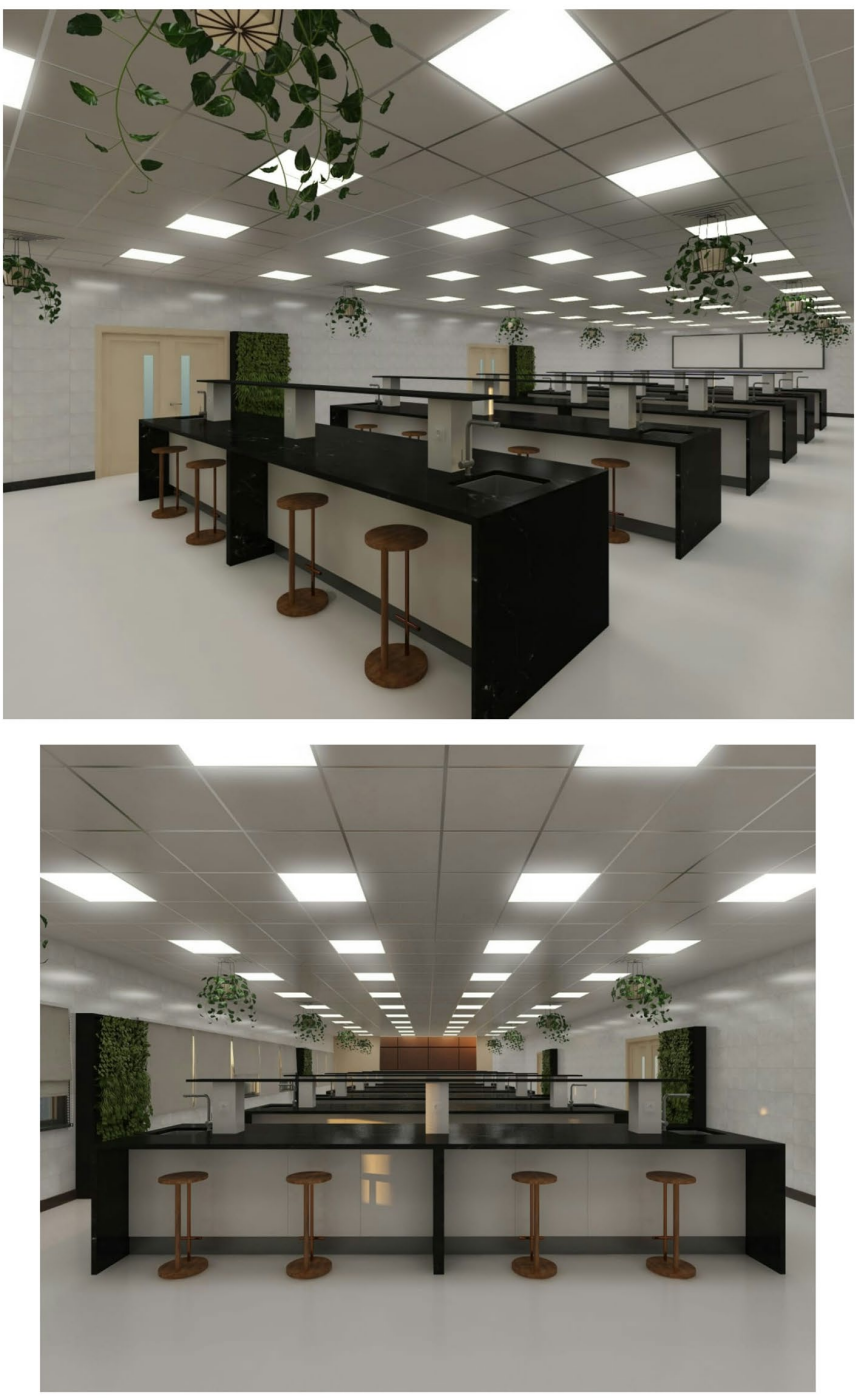

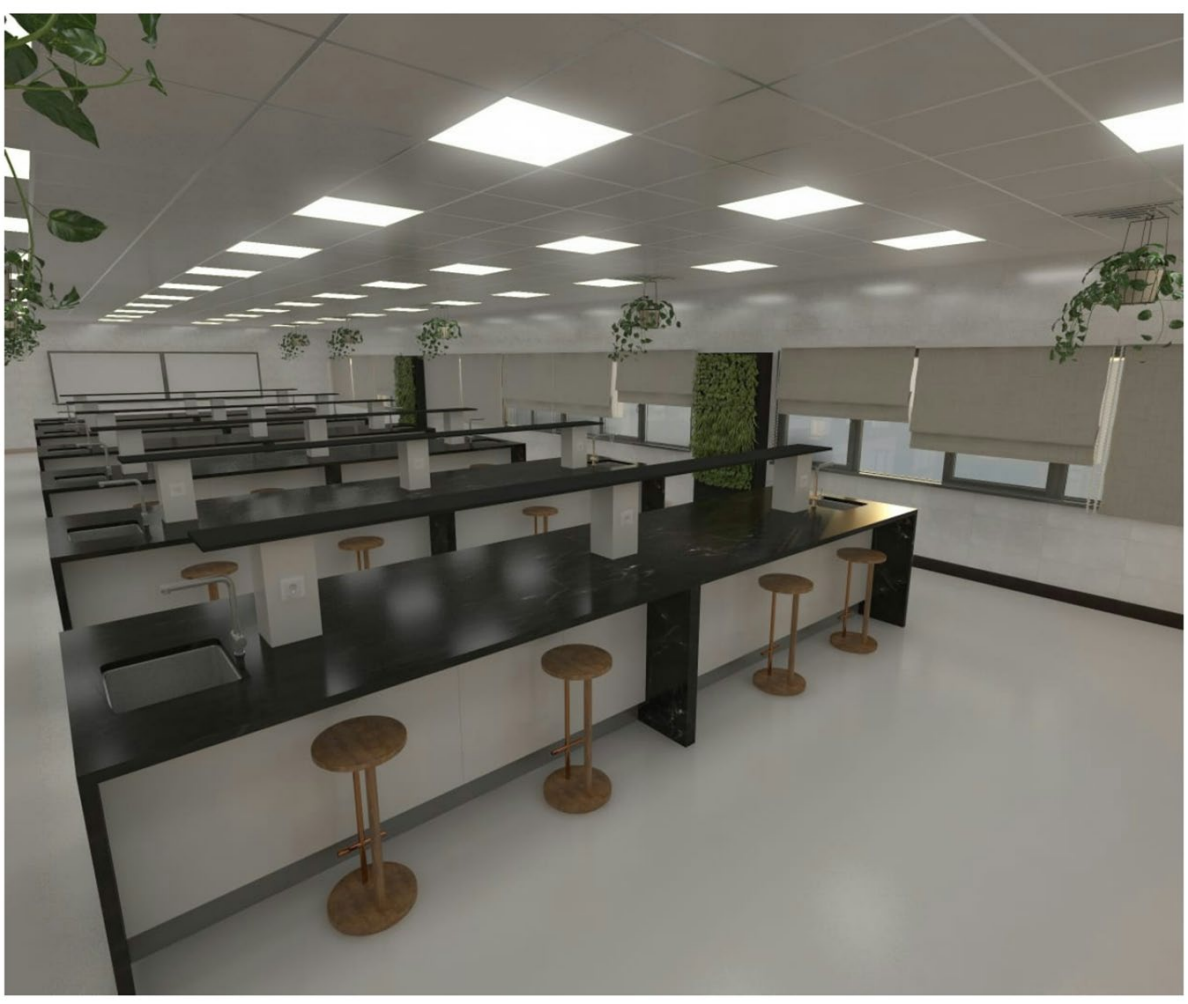
Implementation of plant based biofilters in organic chemistry lab.

In addition to reducing pollutant levels, our results are consistent with the broader benefits of indoor plants on human health and wellbeing. Han et al ^35^ reported through meta-analyses that indoor plants significantly improved physiological, cognitive, and behavioral functions, such as lowering diastolic blood pressure and enhancing academic achievement. Taken together, these findings highlight that *Cordyline fruticosa* was the most effective plant for lowering VOC levels, followed by *Syngonium podophyllum*, *Epipremnum aureum*, and *Chlorophytum comosum*. Although exposure risks for benzene and toluene were found to be within acceptable EPA thresholds, the absence of direct baseline measurements in the control scenario limits our ability to attribute these outcomes solely to ventilation or PPE practices. Rather, the findings suggest that a combination of existing laboratory safety measures and the additional use of plant-based biofilters collectively supported acceptable exposure conditions Incorporating these species as part of plant-based biofilter systems in high-risk environments such as organic laboratories can substantially reduce exposure to hazardous VOCs, improve IAQ, and ensure safer, healthier indoor environments for students, staff, and other occupants (Fig. 12).

### Limitations

This study provides valuable insights into the phytoremediation potential of four ornamental plant species and their contribution to improving indoor air quality; however, several limitations should be acknowledged. The experiments were conducted under controlled laboratory conditions, which may not fully replicate the variability of real-life environments such as differences in ventilation rates, humidity, temperature, and occupancy patterns. The assessment focused only on benzene and toluene, leaving out other potentially harmful VOCs like xylene and formaldehyde, and the monitoring was limited to short-term exposure, which may not reflect long-term dynamics or seasonal variations. Finally, the health risk assessment relied on standard U.S. EPA exposure factors without accounting for individual variability in susceptibility or the protective effects of consistent PPE use, which may influence the accuracy of the estimated cancer and non-cancer risks.

## Conclusion

This study provides new evidence on the application of plant-based biofiltration as a supplementary strategy for improving indoor air quality in an operational organic laboratory environment. The findings demonstrate that exposure to benzene and toluene remained within acceptable limits according to U.S. EPA guidelines under the studied conditions, while the introduction of selected ornamental plant species (*Cordyline fruticosa*, *Syngonium podophyllum*, *Epipremnum aureum*, and *Chlorophytum comosum*) was associated with a measurable reduction in total VOC concentrations and improved indoor air quality indicators. Among the evaluated species, the combined morphological and physiological traits of the plants suggest a notable contribution to pollutant attenuation, highlighting their functional role as passive biofilters in laboratory settings.

A key novelty of this work lies in integrating plant-based phytoremediation within a real operational laboratory context combined with human exposure risk assessment, rather than relying solely on controlled chamber experiments. This approach provides a more applied perspective on the effectiveness of indoor plants under realistic working conditions.

However, it is important to acknowledge that VOC assessment was based on total VOC measurements rather than compound-specific analytical resolution, and baseline exposure estimations were derived through risk assessment modeling rather than continuous direct monitoring. Furthermore, the relatively short experimental duration and focus on only two target compounds (benzene and toluene) limit broader generalization.

Overall, the results support the potential of indoor ornamental plants as complementary elements in integrated risk management frameworks, alongside ventilation systems and personal protective equipment. Future research should focus on long-term monitoring, compound-specific VOC profiling, and multi-pollutant assessment to further validate and optimize the role of phytoremediation in occupational and laboratory air quality management.

## Recommendations

Regular monitoring of VOC levels should be conducted to assess the effectiveness of plant-based interventions and make necessary adjustments to plant selection and placement. Employers should implement a combination of natural and mechanical air purification strategies, including increased ventilation, proper PPE usage, and indoor plants, to mitigate health risks associated with VOC exposure. Periodic health risk assessments should be conducted to evaluate the impact of VOC exposure on laboratory personnel and adjust mitigation strategies accordingly. Further research should investigate the long-term performance of plant-based biofilters under varying laboratory conditions, including temperature, humidity, and light intensity. The development of smart, sensor-based monitoring systems integrated with phytoremediation technologies could provide real-time air quality data and optimize plant-based VOC removal efficiency. By adopting these recommendations, pharmaceutical laboratories can effectively enhance air quality, reduce occupational health risks, and promote a sustainable, eco-friendly approach to pollution control.

## Data Availability

The datasets used and/or analyzed during the current study are available from the corresponding author on reasonable request.
